# Natural language processing (NLP)-assisted assessment of neurologic severity and functional outcomes in factor Xa inhibitor–related intracranial hemorrhages

**DOI:** 10.3389/fneur.2026.1750864

**Published:** 2026-05-18

**Authors:** S. Scott Sutton, Joseph Magagnoli, Ryan Pittman, Tammy H. Cummings, Richard Ofori-Asenso

**Affiliations:** 1 Department of Clinical Pharmacy and Outcomes Sciences, College of Pharmacy, University of South Carolina, Columbia, SC, United States; 2 Dorn Research Institute, Columbia VA Healthcare System, Columbia, SC, United States; 3 BioPharmaceuticals Medical, AstraZeneca, Cambridge, United Kingdom

**Keywords:** factor Xa inhibitors, functional outcome, ICH, intracranial hemorrhage, modified rankin scale, MRS, natural language processing, NLP

## Abstract

**Introduction:**

Factor Xa inhibitors (FXai) are associated with a risk of serious bleeding, including intracranial hemorrhage (ICH), which can lead to long-term disability. Functional status, often measured using the modified Rankin Scale (mRS), is a key outcome after ICH but is rarely recorded in structured electronic medical record (EMR) fields. This study used natural language processing (NLP) in the retrospective derivation of mRS scores from unstructured clinical notes and evaluate clinical correlates of functional outcomes in a national cohort of US veterans with FXai-related ICH.

**Methods:**

This retrospective cohort study used data from the Veterans Affairs (VA) Informatics and Computing Infrastructure (VINCI) from March 2014 to May 2022. Adults (≥18 years) hospitalized for direct or indirect FXai-related ICH and treated with reversal or replacement agents were included. Functional outcomes were assessed using mRS scores derived via NLP of unstructured clinical notes. Glasgow Coma Scale (GCS) scores were extracted using an algorithm to support model adjustment and NLP reliability.

**Results:**

The cohort included 269 patients (mean age 75.7 years; 96.7% male). An NLP algorithm was used to retrospectively derive mRS scores, and GCS scores were extracted using an algorithm to support NLP accuracy and model adjustment. Favorable functional status (mRS 0–3) was observed in 36.1% at baseline, increasing to 58.3% at 90 days. Higher baseline mRS and Charlson Comorbidity Index (CCI) were independently associated with worse functional outcomes (mRS ≥ 4) at discharge and 90 days (*P* ≤ 0.039).

**Discussion:**

Retrospective derivation of mRS scores from clinical documentation is feasible using NLP. This approach demonstrates functional outcome assessment in large-scale EMR-based studies when direct measurement is unavailable. Because long-term recovery after ICH is often defined by functional status rather than survival alone, incorporating mRS into retrospective analyses may improve the evaluation of interventions for FXai-related ICH.

## 1 Introduction

Direct and indirect factor Xa inhibitors (FXai) are widely used for stroke prevention and the treatment of venous thromboembolism due to their favorable benefit-risk profile (1). However, as with all anticoagulants, FXai carry the risk of serious bleeding complications, including intracranial hemorrhage (ICH), which is associated with high mortality and substantial long-term disability (2, 3). Functional recovery following ICH varies based on the severity of neurologic injury, timeliness and quality of treatment, comorbidity burden, and access to rehabilitation (4–6).

The modified Rankin Scale (mRS) is a standardized and widely used measure of disability and dependence following neurologic events such as ICH (7). It serves as a primary endpoint in many randomized clinical trials and observational studies assessing functional outcomes (8–11). Despite its clinical importance, mRS scores are rarely captured in structured fields within electronic medical records (EMRs). Instead, functional status is typically embedded within unstructured clinical narratives, such as physician progress notes, discharge summaries, and rehabilitation documentation. This limits the ability to conduct large-scale retrospective analyses of functional outcomes using routine healthcare data.

Natural language processing (NLP) enables structured extraction of clinically meaningful information from free-text documentation and has emerged as a key tool in computational phenotyping. Prior studies have demonstrated the feasibility of using NLP to identify stroke characteristics, classify neurologic events, and extract severity measures from narrative text (12–14). However, the application of NLP to systematically derive functional outcome measures such as the mRS in large EMR-based cohorts remains limited.

In addition to functional status, neurologic severity measures such as the Glasgow Coma Scale (GCS) play a critical role in prognostication following ICH. Unlike mRS, GCS values are more frequently documented explicitly but still require algorithmic extraction from clinical text using text parsing and structured data extraction. This offers an opportunity to validate and incorporate such severity measures into retrospective outcome analyses.

In this study, we applied an NLP-assisted abstraction framework to retrospectively derive mRS scores from unstructured clinical notes in the EMRs of US veterans hospitalized with FXai-related ICH. Baseline functional status was inferred from documentation during the index hospitalization, and follow-up assessments were derived from routine clinical documentation. We also implemented NLP extraction of GCS scores to support neurologic severity adjustment and to validate computational abstraction within this dataset. All patients included were treated with reversal or replacement agents for management of FXai-associated bleeding.

Building on prior work evaluating mortality outcomes in this population (15), the present study extends EMR-based research by enabling systematic assessment of functional outcomes. Because functional status measures such as the modified Rankin Scale (mRS) are rarely captured in structured fields, we implemented an NLP-assisted approach to identify and temporally organize relevant clinical documentation from unstructured notes. This hybrid method combined automated note retrieval with structured expert adjudication to derive clinically meaningful functional outcome measures. By integrating NLP-assisted chart abstraction within a large national VA cohort, this study demonstrates a practical and reproducible framework for incorporating functional outcomes into retrospective neurologic research.

## 2 Materials and methods

### 2.1 Data and study population

This retrospective cohort study used data from the US Department of Veterans Affairs (VA), the largest integrated health care system in the country (16). Individual-level information was obtained from the Veterans Affairs Informatics and Computing Infrastructure (VINCI), including demographics, administrative claims, pharmacy data, and unstructured clinical notes from the Text Integration Utilities [TIU; (17)]. Additional details on data accuracy, completeness, and access methods are available at virec.research.va.gov (search term: VINCI). The study was approved by the VA Institutional Review Board (IRB #1657754) and received Research and Development Committee approval. The study was conducted in compliance with the Department of Veterans Affairs requirements and received Institutional Review Board (1657754) and Research and Development Approval. Informed consent was waived for this retrospective non-observational study.

We identified patients hospitalized with ICH between March 2014 and May 2022, based on International Classification of Diseases (ICD) revision 9, 10 (ICD-9/10) codes indicating bleeding (18, 19). Hemorrhage location was categorized based on the initial site documented in the electronic health record and corresponding ICD diagnosis codes. Multi-compartment bleeds were included but classified according to the primary anatomic location; extension into additional compartments was not modeled separately. Patients were included if they received a reversal or replacement agent (andexanet alfa or 4F-PCC) and had a prescription for apixaban, rivaroxaban, edoxaban, or enoxaparin. Patients with prescriptions for warfarin or dabigatran in the 90 days prior to the bleed were excluded. Andexanet alfa is a US Food and Drug Administration–approved reversal agent for apixaban and rivaroxaban in cases of life-threatening bleeding (20). 4F-PCC, although approved for warfarin reversal, is frequently used off-label for FXai-related bleeds (21). Enoxaparin, an indirect FXai, was included given its similar clinical management to direct oral anticoagulants in trials (eg, ANNEXA-4) and real-world settings (22, 23).

Collected data included patient demographics (age, race, sex), intensive care unit (ICU) stays (identified through ward and transfer location data), and ventilation use (identified via ICD-9/10 and Current Procedural Terminology procedure codes). Concomitant medication use, including agents like vitamin K, was extracted from inpatient pharmacy databases, Bar Code Medication Administration data, and Current Procedural Terminology medication administration codes.

### 2.2 Unstructured data and natural language processing

The VINCI platform includes Text Integration Utilities (TIU) files containing narrative provider documentation. Because modified Rankin Scale (mRS) scores are rarely documented in structured electronic medical record fields, we implemented a hybrid workflow combining natural language processing (NLP)–assisted document retrieval and targeted text extraction with structured expert adjudication.

TIU notes from the index hospitalization through 100 days post discharge were programmatically queried using a predefined lexicon of functional-status–related terms, including “activities of daily living,” “ADL,” “IADL,” “functional status,” “independent,” “assist,” “wheelchair,” “bedbound,” “cognitive status,” and related variants. The NLP component first first identified notes containing relevant terminology and then extracted surrounding text segments (e.g., sentences and adjacent context) describing functional capacity, dependence, mobility, or cognitive impairment. Extracted segments were temporally indexed relative to reversal or replacement therapy administration and organized into prespecified clinical time windows: baseline (peri-treatment), hospital discharge, 30 days post-treatment, and 90 days post-treatment.

The primary outcome, mRS (range 0–6), was not directly documented in structured form and therefore required retrospective abstraction. Two independent reviewers (one PharmD and one one PhD) evaluated the NLP-extracted functional text segments at each time point and assigned mRS scores using predefined abstraction criteria aligned with standard mRS definitions. Reviewers assessed documented independence in activities of daily living, ambulatory status, need for assistance, institutionalization, and cognitive impairment affecting functional independence. Discrepancies in assigned scores were resolved through structured discussion to achieve consensus. This hybrid approach enabled systematic identification and structured presentation of functional documentation while preserving clinical adjudication for final outcome classification. Baseline mRS was defined as functional status during the index hospitalization in temporal proximity to reversal or replacement therapy administration, as inferred from clinical documentation describing early post-ICH functional capacity. Additional clinical variables, including Glasgow Coma Scale (GCS) scores, were extracted using a validated NLP algorithm. Extracted GCS values were incorporated into subsequent analyses examining associations with functional outcomes.

### 2.3 Statistical analysis

Baseline characteristics of the study population were summarized using descriptive statistics. The distribution of mRS scores (0–6) was calculated at 4 time points: baseline, discharge, 30 days post-treatment, and 90 days post-treatment. To identify clinical and demographic factors associated with poor functional outcome, defined as an mRS score ≥4, we performed separate multivariable logistic regressions at each follow-up time point (24–26). Patients with missing data at the follow-up points were excluded. Covariates included: baseline mRS score, baseline GCS score, age, race, sex, FXai type, Charlson Comorbidity Index (CCI), ICU admission, mechanical ventilation, transfusion use, ICH type, and year of admission. In addition, we used ordinary least squares regression to examine associations between these same covariates and mRS score as a continuous variable, to better capture the full spectrum of functional outcomes. Statistical significance was assessed at a 2-sided alpha of 0.05. Analyses were conducted using SAS 9.4 and R.

## 3 Results

**Figure 1:**
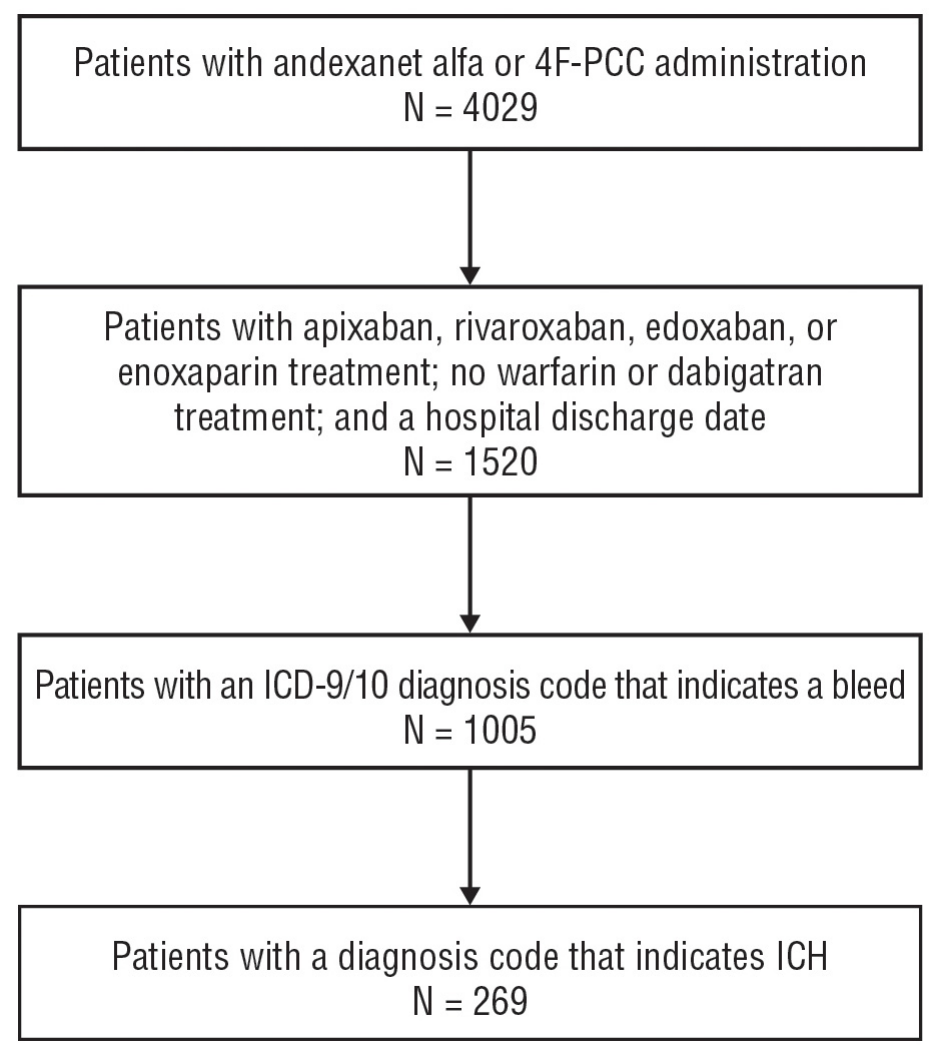
Study participants. 4F-PCC, 4-factor prothrombin complex concentrate; ICD-9/10, International Classification of Diseases, Ninth Ninth and Tenth Tenth Revisions; ICH, intracranial hemorrhage.

Of 4,029 patients that received reversal or replacement therapy during the study period, 269 met inclusion criteria for FXai-related ICH (Figure 1). Of these, 64 (23.8%) were treated with andexanet alfa and 205 (76.2%) with 4F-PCC. The mean age was 75.7 years (SD 10.9), with most patients being male (96.7%) and White (69.9%; Table 1). The mean CCI was 5.8 (SD 3.7), reflecting substantial comorbidity burden. The most common comorbidities included cerebrovascular disease (68.0%), congestive heart failure (50.6%), and diabetes (52.0% without complications; 37.2% with complications). A majority of patients were admitted to the ICU (85.1%), and 14.1% required mechanical ventilation. Subdural hemorrhage was the most common ICH type (42.4%), followed by intraparenchymal (36.4%). The median hospital stay was 6 days. At discharge, 50.6% of patients returned home, while 16.0% were discharged to a nursing home, 13.4% were transferred to another hospital, and 17.5% died in the hospital. Apixaban was the most commonly used FXai (68.0%), followed by rivaroxaban (17.1%), enoxaparin (12.6%), and edoxaban (2.2%).

**Table 1:** Baseline Characteristics.

Characteristic	N = 269
Reversal agent, *n* (%)	
Andexanet alfa	64 (23.8)
4F-PCC	205 (76.2)
15.6-7,-1.3242pt Age, mean (SD)	75.7 (10.9)
Race, *n* (%)	
Black	67 (24.9)
Other/unknown	14 (5.2)
15.6-7,-1.3242pt White	188 (69.9)
Sex, *n* (%)	
Female	9 (3.3)
Male	260 (96.7)
15.6-7,-1.3242pt Hospitalized prior to bleed event, *n* (%)	46 (17.1)
Location of bleed^a^, *n* (%)	
Subarachnoid	26 (9.7)
Intraparenchymal	98 (36.4)
Intraventricular	19 (7.1)
Subdural	114 (42.4)
Unknown	12 (4.5)
15.6-7,-1.3242pt CCI score, mean (SD)	5.8 (3.7)
Comorbidity, *n* (%)	
Myocardial infarction	50 (18.6)
Congestive heart failure	136 (50.6)
Peripheral vascular disease	86 (32.0)
Cerebrovascular disease	183 (68.0)
Dementia	54 (20.1)
Chronic pulmonary disease	98 (36.4)
Connective tissue disease-rheumatic disease	9 (3.3)
Peptic ulcer disease	5 (1.9)
Mild liver disease	38 (14.1)
Diabetes without complications	140 (52.0)
Diabetes with complications	100 (37.2)
Paraplegia and hemiplegia	16 (5.9)
Renal disease	108 (40.1)
Cancer	63 (23.4)
Moderate or severe liver disease	15 (5.6)
Metastatic carcinoma	21 (7.8)
HIV/AIDS	< 5^b^
Drug abuse	60 (22.3)
15.6-7,-1.3242pt Mental health condition	170 (63.2)
DOAC, *n* (%)	
Apixaban	183 (68.0)
Edoxaban	6 (2.2)
Rivaroxaban	46 (17.1)
Enoxaparin	34 (12.6)
Concomitant medications, *n* (%)	
Plasma	6 (2.2)
Tranexamic acid	< 5^b^
Vitamin K	55 (20.4)
Platelets	23 (8.6)
Red blood cells	19 (7.1)
Activated 4F-PCC	15 (5.6)
15.6-7,-1.3242pt ICU stay, *n* (%)	229 (85.1)
Discharge destination, *n* (%)	
Home	136 (50.6)
Hospice	< 5^b^
Nursing home	43 (16.0)
Other hospital	36 (13.4)
Death	47 (17.5)
Missing	< 5^b^
Ventilation, *n* (%)	38 (14.1)
15.6-7,-1.3242pt Transfusion, *n* (%)	23 (8.6)
Year, *n* (%)	
2014–2016	16 (5.9)
2017–2018	41 (15.2)
2019–2022	212 (78.8)

4F-PCC, 4-factor prothrombin complex concentrate; CCI, Charlson Comorbidity Index; DOAC, direct oral anticoagulant; HIV/AIDS, human immunodeficiency virus/acquired immunodeficiency virus; ICU, intensive care unit; SD, standard deviation.

^a^Each patient was assigned a single location based on the predominant site of hemorrhage. Bleeds can span multiple compartments (eg, intraparenchymal hemorrhage with intraventricular hemorrhage extension or subdural hematoma with subarachnoid hemorrhage); therefore, the data represents the primary location of the bleed as documented in electronic health record.

^b^Due to patient privacy, frequencies including < 5 patients cannot be reported.

### 3.1 Functional status

**Figure 2:**
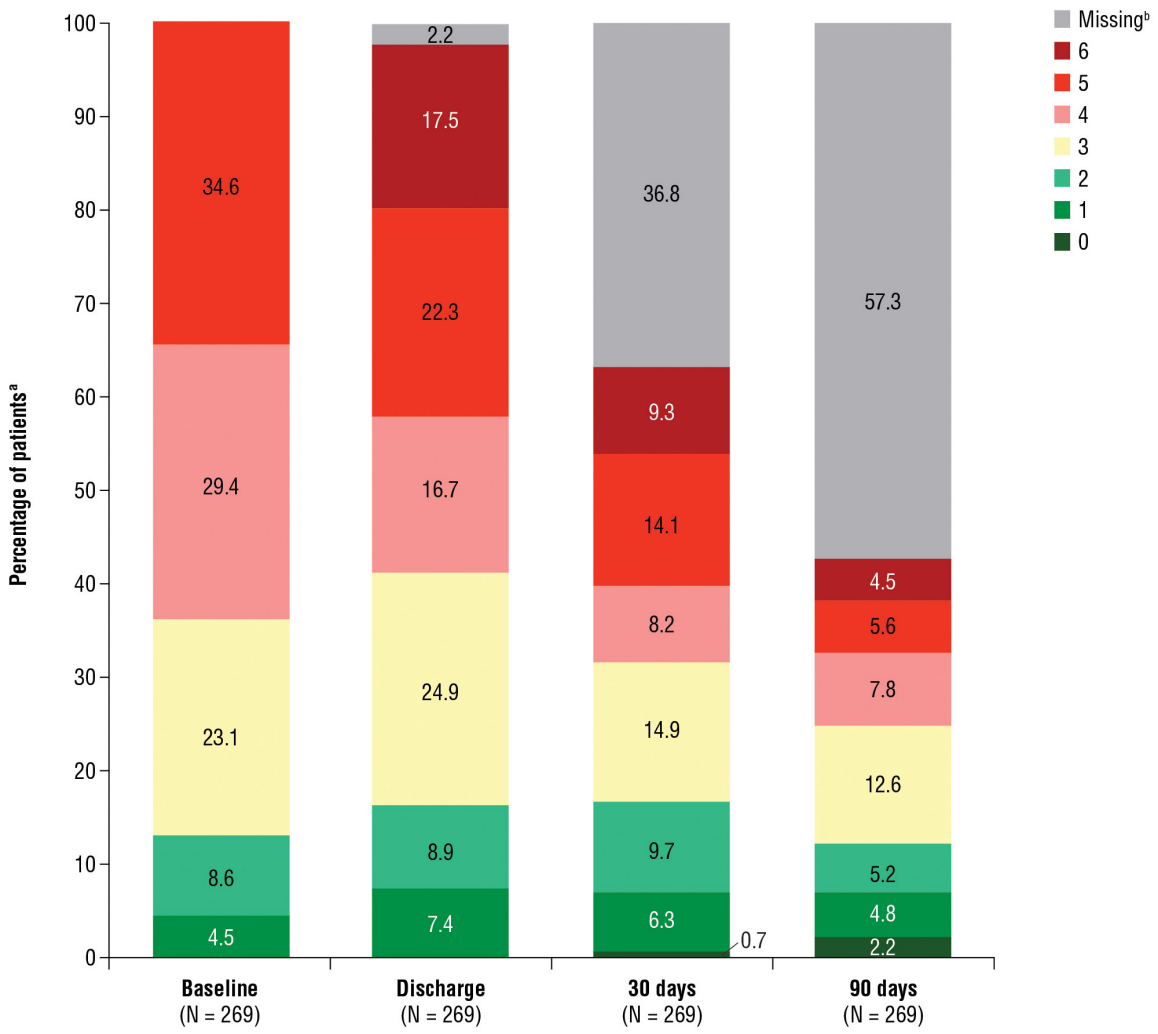
Distribution of mRS scores at baseline, discharge, 30 days, and 90 days. CCI, Charlson Comorbidity Index; mRS, modified Rankin Scale. The patients in this cohort had a CCI >5, which is very high and had the potential to significantly impact the results. ^a^Percentages may not total 100% due to rounding. ^b^Percentages reflect the proportion of patients in each mRS category at each time point. Missing data are shown in gray.

The mean (SD) mRS scores were 3.8 (1.1) at baseline, 3.9 (1.5) at discharge, 3.6 (1.6) at 30 days, and 3.3 (1.6) at 90 days. The proportion of patients with favorable functional status (mRS 0–3) steadily increased over time: 36.1% at baseline, 42.2% at discharge, 50.0% at 30 days, and 58.3% at 90 days (Figure 2). Between baseline and follow-up time points, 55.9% of patients had no change in mRS score at discharge, 44.7% at 30 days, and 40.9% at 90 days (Table 2). Improvements (ie, a lower mRS score compared to baseline) were seen in 16.7%, 28.8%, and 40.0% of patients at those same respective time points. Conversely, functional deterioration (ie, higher mRS) occurred in 27.4% of patients at discharge, 26.5% at 30 days, and 19.1% at 90 days post-treatment. Among patients with non-missing data, the survival rate was 82.1% (216/263) at discharge and 89.6% (103/115) at 90 days postevent.

**Table 2:** Change in functional status from baseline over time.

Score change, *n* (%)	Baseline to discharge (*N* = 263)	Baseline to 30 days (*N* = 170)	Baseline to 90 days (*N* = 115)
No change	147 (55.9)	76 (44.7)	47 (40.9)
Decreased mRS score	44 (16.7)	49 (28.8)	46 (40.0)
Increased mRS score	72 (27.4)	45 (26.5)	22 (19.1)
Unavailable at follow-up, n	6	99	154

mRS, modified Rankin Scale.

In multivariable logistic regression, baseline mRS score was the strongest predictor of poor functional outcome (mRS ≥ 4) at all time points (Table 3). At discharge, older age (odds ratio [OR] 1.05; 95% confidence interval [CI], 1.01, 1.10; *P* = 0.021) and higher CCI score (OR 1.13; 95% CI, 1.01, 1.27; *P* = 0.033) were also significantly associated with worse outcomes. At 30 days, ICU admission was associated with reduced odds of poor outcome (OR 0.13; 95% CI, 0.03, 0.48; *P* = 0.001), while intraparenchymal hemorrhage (vs. subarachnoid) was associated with increased odds of poor outcome (OR 4.92; 95% CI, 1.13, 23.11; *P* = 0.034). At 90 days, baseline mRS and CCI remained significant predictors of poor functional outcomes. When mRS was modeled as a continuous outcome using ordinary least squares regression, baseline mRS score remained a consistent predictor of worse functional status across all follow-ups (Table 4). Lower GCS scores were associated with higher mRS scores at discharge and 30 days. Age and CCI were associated with higher mRS scores at discharge and 90 days. ICU stay was associated with better outcomes at 30 days, and White race was associated with better functional status at 90 days.

**Table 3:** Multivariable logistic regression of poor functional status (mRS ≥4).

Variable	Discharge (*n* = 263)		30 days (*n* = 170)		90 days (*n* = 113)^a^	
	OR (95% CI)	*P* value^b^, ^c^	OR (95%CI)	*P* value^b^, ^c^	OR (95% CI)	*P* value^b^, ^c^
Intercept	0 (0.00, 0.03)	**0.004**	0.02 (0.00, 15.25)	0.232	0 (0.00, 0.00)	**0.002**
Baseline mRS score	8.38 (4.70, 16.53)	**< 0.001**	5.49 (3.09, 10.92)	**< 0.001**	6.34 (3.06, 15.44)	**< 0.001**
Baseline GCS score	0.8 (0.60, 0.99)	0.037	0.92 (0.72, 1.12)	0.403	1.17 (0.91, 1.52)	0.218
15.6-7.4,-1.3498pt Age	1.05 (1.01, 1.10)	**0.021**	1 (0.96, 1.04)	0.987	1.03 (0.98, 1.08)	0.296
Race (reference, Black)						
Other/unknown	2.68 (0.32, 24.78)	0.361	0.25 (0.02, 2.47)	0.240	0.33 (0.03-3.80)	0.372
White	1.3 (0.50, 3.41)	0.582	0.8 (0.29, 2.18)	0.659	0.57 (0.17, 1.86)	0.353
15.6-7.4,-1.3498pt Male	1.66 (0.17, 16.53)	0.658	0.55 (0.04, 8.68)	0.664	0.87 (0.03, 28.49)	0.933
DOAC (reference, apixaban)						
Edoxaban	10.46 (0.68, 158.70)	0.091	4.24 (0.23, 71.43)	0.310	–	–
Enoxaparin	0.68 (0.18, 2.62)	0.579	0.38 (0.09, 1.52)	0.173	1.07 (0.23, 4.88)	0.933
Rivaroxaban	0.39 (0.12, 1.21)	0.104	0.37 (0.10, 1.29)	0.119	0.26 (0.04, 1.34)	0.109
Charlson Comorbidity Index	1.13 (1.01, 1.27)	**0.033**	1.04 (0.92, 1.18)	0.554	1.2 (1.03, 1.41)	**0.016**
ICU stay	1.11 (0.36, 3.34)	0.857	0.13 (0.03, 0.48)	**0.001**	1.11 (0.26, 4.95)	0.886
Ventilation	1.11 (0.19, 6.60)	0.908	1.46 (0.26, 9.04)	0.671	1.73 (0.23, 11.95)	0.577
15.6-7.4,-1.3498pt Transfusion	1.28 (0.31, 5.57)	0.729	1.29 (0.26, 7.29)	0.762	1.12 (0.19, 6.95)	0.904
ICH type (reference, subarachnoid)						
Intraparenchymal	1.08 (0.30, 3.78)	0.906	4.92 (1.13, 23.11)	**0.034**	–	–
Intraventricular	0.71 (0.09, 4.60)	0.723	2.32 (0.18, 24.50)	0.503	–	–
Subdural	1.06 (0.29, 3.82)	0.928	2.71 (0.63, 12.40)	0.184	–	–
15.6-7.4,-1.3498pt Unknown	5.99 (0.46, 169.91)	0.181	2.96 (0.28, 41.39)	0.373	–	–
**Year (reference, 2014–2016)**						
2017–2018	1.27 (0.23, 7.24)	0.782	1.13 (0.17, 7.80)	0.899	1.05 (0.07, 17.21)	0.969
2019–2022	1.53 (0.34, 7.30)	0.582	1.74 (0.35, 9.62)	0.504	3.22 (0.31, 43.86)	0.332

CI, confidence interval; DOAC, direct oral anticoagulant; GCS, Glasgow Coma Scale; ICH, intracranial hemorrhage; ICU, intensive care unit; mRS, modified Rankin Scale; OR, odds ratio.

^a^Patients on edoxaban were excluded from the analysis due to the small numbers resulting in large parameter estimates.

^b^Likelihood ratio test *P* values and 95% CI profiles are reported.

^c^*P* values in bold indicate statistical significance.

**Table 4:** Linear regression of mRS score as a continuous outcome.

Variable	Discharge (*n* = 263)		30 days (*n* = 170)		90 days (*n* = 113)^a^	
	Estimate (95% CI)	*P* value^b^	Estimate (95% CI)	*P* value^b^	Estimate (95% CI)	*P* value^b^
Intercept	−1.11 (−2.75, 0.53)	0.184	1.41 (−1.49, 4.32)	0.337	−0.17 (−3.50, 3.16)	0.92
Baseline mRS score	0.89 (0.77, 1.01)	**< 0.001**	0.77 (0.57, 0.97)	**< 0.001**	0.68 (0.44, 0.93)	**< 0.001**
Baseline GCS score	−0.08 (−0.12, −0.04)	**< 0.001**	−0.09 (−0.17, −0.00)	**0.046**	−0.07 (−0.18, 0.04)	0.224
15.6-7.4,-1.3498pt Age	0.02 (0.01, 0.03)	**< 0.001**	0.02 (−0.00, 0.04)	0.134	0.02 (0.00, 0.04)	**0.044**
Race (reference, Black)						
Other/unknown	−0.09 (−0.63, 0.46)	0.752	−0.56 (−1.58, 0.45)	0.276	−0.83 (−1.92, 0.26)	0.135
White	0.15 (−0.12, 0.41)	0.269	−0.05 (−0.51, 0.41)	0.828	−0.6 (−1.14, −0.06)	**0.03**
15.6-7.4,-1.3498pt Male	0.27 (−0.39, 0.92)	0.425	−0.23 (−1.41, 0.95)	0.698	−0.27 (−2.10, 1.56)	0.77
DOAC (reference, apixaban)						
Edoxaban	0.55 (−0.20, 1.29)	0.148	0.64 (−0.53, 1.80)	0.282	–	–
Enoxaparin	−0.08 (−0.45, 0.28)	0.661	−0.37 (−1.01, 0.27)	0.258	−0.18 (−0.93, 0.57)	0.629
Rivaroxaban	−0.21 (−0.52, 0.10)	0.179	−0.31 (−0.87, 0.25)	0.276	−0.58 (−1.32, 0.17)	0.126
Charlson Comorbidity Index	0.04 (0.01, 0.07)	**0.012**	0.05 (−0.01, 0.11)	0.092	0.11 (0.04, 0.17)	**0.003**
ICU stay	0.18 (−0.14, 0.50)	0.267	−0.93 (−1.52, −0.35)	**0.002**	0.01 (−0.70, 0.72)	0.984
Ventilation	−0.06 (−0.43, 0.32)	0.762	−0.13 (−0.85, 0.59)	0.725	0.11 (−0.71, 0.94)	0.785
15.6-7.4,-1.3498pt Transfusion	−0.02 (−0.43, 0.40)	0.933	−0.07 (−0.81, 0.67)	0.855	0.21 (−0.58, 1.00)	0.603
ICH type (reference, subarachnoid)						
Intraparenchymal	0.22 (−0.18, 0.62)	0.288	0.62 (−0.14, 1.38)	0.107	–	–
Intraventricular	0.04 (−0.51, 0.60)	0.879	0.07 (−1.01, 1.14)	0.904	–	–
Subdural	0.14 (−0.26, 0.54)	0.494	0.41 (−0.33, 1.16)	0.274	–	–
15.6-7.4,-1.3498pt Unknown	0.26 (−0.39, 0.90)	0.434	0.49 (−0.64, 1.62)	0.39	–	–
Year (reference, 2014–2016)						
2017–2018	0.1 (−0.43, 0.64)	0.702	−0.08 (−0.95, 0.79)	0.857	0.22 (−1.02, 1.46)	0.725
2019–2022	0.04 (−0.44, 0.52)	0.875	−0.09 (−0.86, 0.68)	0.826	0.41 (−0.71, 1.53)	0.467

CI, confidence interval; DOAC, direct oral anticoagulant; GCS, Glasgow Coma Scale; ICH, intracranial hemorrhage; ICU, intensive care unit; mRS, modified Rankin scale.

^a^Patients on edoxaban were excluded from the analysis due to the small numbers resulting in large parameter estimates.

^b^*P* values in bold indicate statistical significance.

## 4 Discussion

In this study, we leveraged NLP to extract clinical information from EMRs and describe functional outcomes in US veterans with FXai-related ICH. Mortality was consistent over time; the proportion of patients alive was approximately 82% at discharge and 90% at 90 days postevent. Functional disability was common at baseline (64% had mRS 4–5), and although improvement occurred over time, many patients remained dependent. Factors associated with greater odds of functional disability included higher baseline mRS score, higher CCI, and—in some models—a history of ICU admission or specific bleed types.

Previous studies have demonstrated that NLP tools can effectively abstract functional measures, such as the mRS, from unstructured clinical notes with acceptable reliability (27–29). NLP also shows promise for predicting long-term outcomes after acute ischemic or hemorrhagic stroke (12–14). For example, Isenberg et al. (28) reported good concordance between mRS scores derived from EMRs and those obtained from in-person assessments, and the Greater Cincinnati/Northern Kentucky Stroke Study showed strong agreement between EMR-derived and telephone-assessed mRS scores at 3 and 6 months (27). However, manual abstraction of mRS scores remains labor-intensive, and the subjective nature of the mRS, particularly in the mid-range, limits reproducibility (30, 31). Additionally, mRS documentation is often absent from clinical notes, especially outside neurology or stroke services. In our study, despite efforts to use a modified version of an existing NLP, mRS could not be reliably extracted in a fully automated fashion due to sparse explicit documentation. Instead, reviewers applied standardized criteria to assign scores based on available clinical documentation. mRS scores were assigned using a standardized abstraction framework aligned with established mRS definitions. Reviewers independently evaluated documented functional status within each predefined time window, with emphasis on independence in activities of daily living (ADLs/IADLs), ambulatory status, need

for assistance or supervision, institutionalization, and cognitive impairment affecting functional independence. For each time point, the most temporally relevant documentation was prioritized, and in cases of uncertainty, assessments were jointly reviewed using predefined criteria to ensure consistent scoring. This manual step introduces the potential for misclassification and precludes traditional validation approaches such as comparison to in-person scoring. To address this limitation, we leveraged the GCS as a complementary measure of neurologic severity. In contrast to mRS, GCS scores were frequently documented explicitly in the EMR and were extractable using an NLP algorithm with minimal human review. GCS values were extracted from clinical documentation using a rule-based approach and compared with manual review to assess concordance. Because extraction relied on documented values within unstructured text, residual variability in value capture may introduce measurement error and could influence the precision of severity adjustment in multivariable models. Although functional independence (mRS 0–2) is frequently used as a threshold in ischemic stroke trials, ICH cohorts often demonstrate greater baseline severity and disability. Accordingly, several contemporary ICH studies have dichotomized outcomes as mRS 0–3 vs. 4–6 to distinguish ambulatory patients with moderate disability from those with severe disability or death. Given the advanced age and comorbidity burden of our cohort, we selected mRS 0–3 to reflect clinically meaningful recovery in this population (10). However, alternative thresholds, such as mRS 0–2 (functional independence), may yield different distributions of favorable outcomes and potentially different associations with clinical predictors. Because recovery after ICH exists along a continuum of disability, results should be interpreted within the context of the selected outcome definition. Importantly, we were able to validate the GCS NLP against manual extraction, supporting its accuracy and reliability. Beyond serving as a surrogate for clinical severity, GCS scores provided 3 key advantages: (1) enabled validation of NLP methodology in this dataset, (2) available in a semi-structured format for most patients, and (3) could be incorporated into multivariable models to control baseline severity. The integration of NLP-derived GCS strengthened the internal validity of our findings and offers a promising methodological framework for future retrospective studies.

Our study also reinforces prior findings on functional trajectory after ICH. The mean mRS score declined over time, and fewer patients experienced worsening functional status as follow-up progressed. These patterns are consistent with previous literature suggesting that patients often recover or plateau following ICH, with few deteriorating after the acute phase (32). Because baseline mRS reflects early post-ICH functional impairment rather than pre-hemorrhage status, its strong association with later outcomes likely represents the prognostic importance of early neurologic injury and functional trajectory. This should not be interpreted as independent causal prediction but rather as early severity characterization. The interpretation of long-term outcomes must also account for attrition and competing risks. Missing data increased substantially at 30 and 90 days (37% and 57%, respectively), likely reflecting mortality, transfer of care, or lack of documented follow-up. Because this retrospective study derived mRS scores from routine clinical documentation rather than protocol-mandated follow-up assessments, 90-day data were incomplete and dependent on real-world encounter patterns within the VA system. This may introduce selection bias and limit the generalizability of the 90-day findings. Mortality was comprehensively captured within the VA system, and patients who died were coded as mRS = 6; therefore, missing follow-up data do not represent unobserved deaths. However, mRS ascertainment at 30 and 90 days was dependent on the presence of routine clinical documentation among survivors. Follow-up at 90 days was dependent on the presence of routine clinical documentation, and patients with available data may differ systematically from those without documented encounters. This introduces the potential for selection and survivorship bias, which may influence the observed distribution of favorable outcomes at later time points. As such, 90-day results should be interpreted as conditional on observed follow-up rather than representative of the full cohort. Because follow-up documentation may be associated with healthcare utilization or functional trajectory, selection bias among surviving patients cannot be excluded. These factors limit causal interpretation of longitudinal changes in functional status. The cohort was medically complex, with a high mean CCI and substantial burden of cardiovascular and mental health comorbidities. In such populations, functional trajectory may be influenced not only by ICH and its treatment but also by underlying frailty, chronic disease progression, and non-neurologic competing risks. Although our multivariable models adjusted for baseline mRS and comorbidity burden, residual confounding related to time-varying health decline cannot be excluded. Comorbidity burden (CCI) and neurologic severity (GCS) were included to capture distinct aspects of baseline risk; however, these variables may be correlated in an older, medically complex population. Potential collinearity could influence the stability and precision of regression estimates, and independent effects of individual covariates should be interpreted with caution. Therefore, observed changes in mRS likely reflect a composite of ICH recovery, treatment response, and background geriatric functional trajectory rather than the isolated effect of reversal or replacement therapy. Additional limitations include the modest sample size, which may limit statistical power, and reliance on retrospective chart review, where completeness and documentation quality may vary. Generalizability is limited given the predominantly older, male veteran population, reflecting the VA population, which limits generalizability to female patients. Because bleed type was classified by initial location rather than explicit modeling of multi-compartment extension, residual confounding related to hemorrhage complexity cannot be excluded. The NLP approach relied primarily on standardized clinical terminology describing activities of daily living and cognitive status, which is less likely to vary by gender. Nonetheless, documentation patterns and recovery trajectories may differ in more sex-diverse populations, and external validation may be necessary to determine whether these results remain consistent in other populations. Formal inter-rater reliability statistics were not calculated prior to consensus adjudication, which limits quantification of scoring variability. Although alternative functional measures such as the Functional Independence Measure (FIM) were explored, documentation was inconsistent. The temporal clustering of cases after 2018 may reflect increased utilization of reversal therapies following the approval of andexanet alfa. Because inclusion required administration of reversal or replacement agents, evolving treatment patterns may have influenced case distribution. However, the NLP extraction framework was applied uniformly across all study years. We were unable to assess system-level changes in formulary policy or documentation practices within the VA during this period. Furthermore, administrative claims data, while useful for identifying exposures and diagnoses, depend on coding accuracy and may lack clinical nuance.

The observed association between ICU admission and lower odds of poor functional outcome should not be interpreted as evidence of a protective effect of ICU care. ICU admission is typically a marker of illness severity; however, in observational datasets, triage patterns and early goals-of-care decisions may result in selection of patients perceived as salvageable for intensive care. Despite adjustment for baseline mRS and GCS, residual confounding related to hemorrhage complexity and treatment intensity likely persists. Despite these limitations, our findings offer important insights into functional recovery following FXai-related ICH and demonstrate the utility of NLP in augmenting EMR-based research. The integration of GCS provides a methodological advancement by allowing validation and adjustment for neurologic severity, addressing a key challenge in retrospective stroke outcomes research. Future studies should incorporate both GCS and mRS to enhance comparative effectiveness research in anticoagulation reversal.

### 4.1 Conclusion

This study demonstrated that NLP can be used in the derivations of retrospectively collected mRS scores from electronic medical records to evaluate functional outcomes following FXai-related ICH. Among US veterans with high comorbidity, functional disability was common at baseline and persisted through 90 days. Higher baseline mRS, ICU stay, and greater comorbidity were associated with poor outcomes. Because mRS scores were infrequently documented, we also extracted GCS scores, which were more consistently recorded and allowed us to demonstrate the feasibility and reliability of our NLP extraction process. Although GCS and mRS measure different outcomes, the successful NLP-based extraction of GCS supports the broader applicability of these methods to clinical text and strengthens confidence in our approach to deriving functional outcome scores.

## Data Availability

The data analyzed in this study is subject to the following licenses/restrictions: These analyses were performed using data that are available within the US Department of Veterans Affairs secure research environment, the VA Informatics and Computing Infrastructure (VINCI). All relevant data outputs are within the manuscript and related tables. Information about data from the Department of Veterans Affairs VINCI can be reviewed at https://www.virec.research.va.gov.
